# Adherence With Online Therapy vs Face-to-Face Therapy and With Online Therapy vs Care as Usual: Secondary Analysis of Two Randomized Controlled Trials

**DOI:** 10.2196/31274

**Published:** 2021-11-03

**Authors:** Sonia Lippke, Lingling Gao, Franziska Maria Keller, Petra Becker, Alina Dahmen

**Affiliations:** 1 Department of Psychology and Methods, Jacobs University Bremen Bremen Germany; 2 Dr. Becker Klinikgruppe Cologne Germany

**Keywords:** psychotherapeutic aftercare, medical rehabilitation, online therapy, face-to-face therapy, care as usual, retention, dropout

## Abstract

**Background:**

Adherence to internet-delivered interventions targeting mental health such as online psychotherapeutic aftercare is important for the intervention’s impact. High dropout rates limit the impact and generalizability of findings. Baseline differences may be putting patients at risk for dropping out, making comparisons between online with face-to-face (F2F) therapy and care as usual (CAU) necessary to examine.

**Objective:**

This study investigated adherence to online, F2F, and CAU interventions as well as study dropout among these groups and the subjective evaluation of the therapeutic relationship. Sociodemographic, social-cognitive, and health-related variables were considered.

**Methods:**

In a randomized controlled trial, 6023 patients were recruited, and 300 completed the baseline measures (T1), 144 completed T2 (retention 44%-52%), and 95 completed T3 (retention 24%-36%). Sociodemographic variables (eg, age, gender, marital status, educational level), social-cognitive determinants (eg, self-efficacy, social support), health-related variables (eg, depressiveness), and expectation towards the treatment for patients assigned to online or F2F were measured at T1.

**Results:**

There were no significant differences between the groups regarding dropout rates (χ^2^_1_=0.02-1.06, *P*≥.30). Regarding adherence to the treatment condition, the online group outperformed the F2F and CAU conditions (*P*≤.01), indicating that patients randomized into the F2F and CAU control groups were much more likely to show nonadherent behavior in comparison with the online therapy groups. Within study groups, gender differences were significant only in the CAU group at T2, with women being more likely to drop out. At T3, age and marital status were also only significant in the CAU group. Patients in the online therapy group were significantly more satisfied with their treatment than patients in the F2F group (*P*=.02; Eta²=.09). Relationship satisfaction and success satisfaction were equally high (*P*>.30; Eta²=.02). Combining all study groups, patients who reported lower depressiveness scores at T1 (T2: odds ratio [OR] 0.55, 95% CI 0.35-0.87; T3: OR 0.56, 95% CI 0.37-0.92) were more likely to be retained, and patients who had higher self-efficacy (T2: OR 0.57, 95% CI 0.37-0.89; T3: OR 0.52, 95% CI 0.32-0.85) were more likely to drop out at T2 and T3. Additionally, at T3, the lower social support that patients reported was related to a higher likelihood of remaining in the study (OR 0.68, 95% CI 0.48-0.96). Comparing the 3 intervention groups, positive expectation was significantly related with questionnaire completion at T2 and T3 after controlling for other variables (T2: OR 1.64, 95% CI 1.08-2.50; T3: OR 1.59, 95% CI 1.01-2.51).

**Conclusions:**

While online interventions have many advantages over F2F variants such as saving time and effort to commute to F2F therapy, they also create difficulties for therapists and hinder their ability to adequately react to patients’ challenges. Accordingly, patient characteristics that might put them at risk for dropping out or not adhering to the treatment plan should be considered in future research and practice. Online aftercare, as described in this research, should be provided more often to medical rehabilitation patients.

**Trial Registration:**

ClinicalTrials.gov NCT04989842; https://clinicaltrials.gov/ct2/show/NCT04989842

## Introduction

### Background

Internet-delivered, online interventions provide many advantages for the prevention and treatment of psychological problems and mental health disorders such as depression, anxiety, and functional limitation [1-3]. Psychotherapeutic online interventions have been shown to be effective [4,5]. However, 1 of 2 patients drop out from many online intervention studies [6]. Dropout limits the impact of these interventions and the generalizability of the findings [7]. Besides, few studies have compared a synchronous online therapy group guided by a therapist with a control group in a face-to-face (F2F) format using the same therapeutic concept (“Curriculum Hannover”) and with a treatment/care as usual (CAU) group (eg, [8]).

So far, 1 study of the few pre-existing studies found an advantage of online therapy over CAU and about the same effects as the F2F format [9]. This research aimed to use the data from this previous research for a secondary data analysis to further investigate, within the German Pension Insurance's framework concept for rehabilitation therapy [10], adherence to the assigned treatment arm and patient dropout and for subjective evaluation of relationship, success, and satisfaction.

Adherence is a key concept [11,12] and is conceptualized as adhering to the assigned treatment (within this study) such as CAU. On the contrary, nonadherent behavior means that patients find themselves a (different) therapy to which they were not assigned: Nonadherence in the CAU arm means that patients registered in an F2F or online treatment format. Nonadherence in the F2F arm would mean that they chose another treatment outside the Curriculum Hannover treatment scope. Nonadherence in the online groups would mean that patients changed to a treatment other than online Curriculum Hannover. Consequences of the lack of instant availability of psychotherapists [13] and psychotherapy were found in terms of patients being nonadherent and joining other kinds of treatments such as inpatient psychotherapeutic treatment, drug therapy, or self-help groups. In a systematic review, lack of time was clearly related with lower adherence [14].

Dropout from the study in all study arms was conceptualized as not completing the questionnaire anymore because the patient intentionally left the study or was not reachable anymore for further measurements [6,7].

It is important to understand why patients display intervention adherence and study retention (as opposed to dropping out [3,6-8]): Different factors can predict whether patients remain in the assigned therapy and the study in general. If we know about such predictors, we can address them so that the program is nurturing the patients’ needs better and to prevent dropout and nonadherence, with the resulting loss of intervention efficacy and effectiveness [5]. This can also improve the impact of the treatments [4]. Accordingly, in the following, the evidence regarding potential correlates and predictors are summarized to explain our study’s design described in the methods section.

### Prior Work on Study Dropout and Adherence

Expansive knowledge already exists on factors affecting study participants, the likelihood of questionnaire completion (eg, [6]), and adherence to the assigned treatment. For instance, in their systematic review of 33 randomized controlled trials (RCTs), Brown et al [11] identified the following reasons for low adherence levels: time issues, little or no interest of the participants, the perception of the participants that treatment is not needed at all or anymore, or the intervention is not effective. They also identified technical problems and other priorities in daily life including holidays and work. Moreover, dissatisfaction with the assigned group was shown to be important. Furthermore, health issues and a fading motivation to participate in the program were also found [11]. The authors also revealed no statistical relationship between the intended duration of the program and adherence with the intervention.

However, a direct comparison of completers in the different intervention arms such as online vs F2F is rare. Further, evidence regarding what drives patients to adhere to the assigned therapy is scarce within internet-delivered psychotherapeutic interventions. A previous study with students [12] investigated counseling delivered online or F2F versus a placebo treatment. Lack of motivation, dissatisfaction with the counseling process, and perceptions that the counselor would not understand students via this medium were all more prominent in online counseling than in F2F counseling [8,12]. However, physical appearance was indicated as a barrier in the F2F group but not in the online group [12]. Moreover, F2F counseling was perceived as excessively straining in light of other duties [12].

### Advantages of Online Interventions

Past studies [4,5] have consistently found that online treatments can save the therapists time and support relapse prevention after F2F therapy. Additional strengths of online interventions over F2F interventions are that they are deliverable from remote locations, need less time commitment, and provide more flexibility for therapists and patients. Another advantage may be that the risk of stigma due to a mental disorder and seeking treatment is reduced [4]. This can overcome the problems with F2F therapies, which furthermore are often not readily available in all regions and where they are needed, resulting in patients promptly starting with their online intervention instead of waiting a long time (which is typical for F2F therapies due to limited availability of therapists [13]). Moreover, patients in psychotherapeutic interventions may miss their F2F sessions or drop out of therapy because they feel as if the location of the therapy is too far away [15]. Thus, online mental health interventions can bridge the gap between patients and therapists when the patient cannot travel to the intervention site (eg, [7]) or both are limited in their mobility.

### Disadvantages of Online Interventions

Online therapies may harbor weaknesses like the requirement of knowledge and skills such as computer and internet health literacy and general literacy [4]. Not every patient may benefit from online psychotherapy or blended therapy forms (ie, a combination of F2F psychotherapy with online interventions modes) due to limited introspection capabilities or the nature of their disorder (ie, severe disorders, chronic syndromes, or personality disorders [13]). Hence, a personalized tool may be needed to consider individual patient characteristics [4]. Traditional therapy settings can help patients with self-reflection, especially if they are not well experienced with expressing their cognitions and emotions [4]. Additionally, online therapies may prevent counselors from reacting to emergency situations like acute psychic decompensation or acute psychosis as adequately as they could in an analogue situation [4]. Negative experiences with digital psychotherapeutic interventions could have the consequence of patients feeling less motivated, feeling unsure, or even avoiding trying F2F therapy. Conversely, relative to F2F interventions, online interventions might have limitations such as higher dropout (eg, 43% computer-guided or online vs 24% clinician-guided or F2F [13]). However, the opposite pattern was found in studies (ie, lower dropout in online interventions [4]). Thus, this needs more systematic investigation.

### Sociodemographic Correlates

In some studies, age was found to be related to the willingness to participate and remain in online research [14], with younger individuals having a higher likelihood of participation in general, but also dropping out easier, than older individuals [16,17].

Women are typically more likely to participate in surveys (eg, [12]), but this is often reversed when internet studies are reviewed (eg, [16]). It is explained by the finding that men have, on average, more favorable attitudes, beliefs, and self-efficacy expectations toward technology use [18]. With that, men are more likely to remain in some online intervention studies, as indicated by a higher completion rate of modules compared with women [14], and more likely to drop out in other studies (eg, [19]).

Marital status, social integration, and social support are helpful for retaining patients in online interventions [8,14,17] and to mediate the intervention effect on symptomatology [7,16]. Study participants with low social support were more likely to seek such social support or consumer feedback in treatments [14,20]. Studies also demonstrated that individuals who heard about the online treatment from a family member and those with social stress were more likely to be strained and to drop out from the treatment. Dropout for these individuals was largely due to family responsibilities such as caring for a child or another family member [8,17,20].

The typical finding regarding educational level is that more literate participants harbor a higher willingness to participate in and are more successful at remaining in the study [20,21]. On the contrary, individuals with poor education, low income, and higher risk for unemployment were more likely to not make use of health care or to not benefit from it compared with their more privileged counterparts [16,17]. However, an aggregation of only online psychological interventions revealed no conclusive evidence regarding employment [14].

### Health-Related, Psychosocial, and Social-Cognitive Variables

Regarding health-related and psychosocial variables, along with workability, the typical pattern is that the more somatoform complaints, depressive symptoms, and phobic anxiety, the lower their mental well-being; in addition, the more interactional difficulties patients have, the more likely they are to drop out [6,17,20].

Regarding social-cognitive variables, self-efficacy was found not to be directly related to adherence but to planning, which predicted adherence [3,14]. Finally, higher treatment expectancy regarding the treatment efficacy was related to greater study retention and adherence because improvement expectation also helped participants overcome difficulties and remain motivated [14,20]. These findings can be explained using the theoretical backdrop of the health action process approach (HAPA, [22]), which will also be used for this study. In the study by Zarski et al [3], 14% of the variance in treatment adherence could be explained by variables in the HAPA model; however, it remains unknown whether other characteristics beyond the HAPA variables can explain more variance in study dropout.

### Therapeutic Relationship

For any form of psychosomatic rehabilitation aftercare led by a therapist, establishing a therapeutic relationship is of tremendous importance [7]. To successfully provide online therapies and build a therapeutic relationship, therapists require training, induction, clear guidelines, in-depth information and training, and continuing education and training [23].

This leads to the following question: If the therapists are well-trained in computer literacy and internet skills, will satisfaction with the relationship and patient progress in therapy be equal in the online therapy and the F2F treatment?

### Goal of This Study

Due to the various sociodemographic variables, social-cognitive determinants, and health-related variables influencing adherence to online and F2F-therapies, we need to consider whether baseline differences may be putting patients at risk for dropping out.

This study made use of a setting with medical rehabilitation and aftercare [10]. Medical rehabilitation for psychosomatic patients in Germany is provided in clinics, where disadvantages of online interventions can also be addressed to enable patients and therapists as well to use online psychosomatic aftercare. For instance, technical requirements can be cleared, and affinity for internet use (ie, digital health literacy) can be trained to ensure patient-treatment fit [8].

However, so far, no study can be found addressing the comparison of the same aftercare delivered online (content and procedure of the therapy; Curriculum Hannover [24,25]) with F2F and CAU in terms of study dropout and intervention withdrawal. Thus, this was the main aim of this study, since the usefulness of Curriculum Hannover was tested before and clearly revealed its superiority to CAU, as well as parity between the internet and F2F delivery modes [9].

### Research Questions

We aimed to answer the following research questions: (1) What difference exists between online therapy groups (ONL1 [equivalence study], ONL2 [superiority study]) and control groups (F2F, CAU) regarding patients who complete the questionnaires (completers) and patients who adhere to the assigned therapy? (2) What reasons do patients have for not adhering with the assigned therapy? (3) What factors are related with completing the different study arms at T2 and T3? (4) Does the improvement expectation differ between the online therapy groups (ONL1, ONL2) and the control groups (F2F, CAU), and does it relate to questionnaire completion at T2 (9 [superiority study] or 12 [equivalence study] months after the end of the rehabilitation) and T3 (15 [superiority study] or 18 [equivalence study] months after the end of rehabilitation) after controlling for other variables? (5) Does the subjective evaluation of the online psychotherapy (ONL1, ONL2) and F2F psychotherapy differ with regard to relationship satisfaction, success satisfaction, and satisfaction with therapy?

## Methods

### Ethical Considerations

All participants were informed about the purpose of the study (including information on the length of the questionnaires and data storage procedures) via a participant information form and an informed consent form (all forms can be seen in the appendix to the CONSORT eHealth statement [9]). All procedures conducted in this study were in accordance with the ethical standards of the 1964 Helsinki declaration and its later amendments or comparable ethical standards.

The questionnaire prior to the intervention was mandatory for every study participant to avoid missing units. However, whether study participants actually answered the individual questions was voluntary. In the case of questions from the patient, a project manager was at hand to reduce the risk of dropout from the study. The study protocol was published together with the primary results [9].

The study protocol was approved by the Ethics Committee of the North Rhine Medical Association (Ärztekammer Nordrhein; No. 2015351; Dec 4, 2015). As this study was run in a rehabilitation treatment and aftercare setting, approval was given by the pension funds (Bund, Braunschweig-Hannover and Rheinland) and corresponding patient councils [9]. The clinical trial was retrospectively registered with ClinicalTrials.gov (NCT04989842).

### Recruitment

We recruited 6023 patients at their psychosomatic rehabilitation clinic. After excluding noneligible patients, 300 completed the baseline measures (T1). All rehabilitants who had participated in a psychotherapeutic rehabilitation treatment with Dr. Becker Klinik Möhnesee (March 2017 to May 2018), Dr. Becker Klinik Juliana (March 2017 to April 2018), or Dr. Becker Burgklinik (complete period between March 2017 and September 2020) were eligible for the aftercare therapy offered following the medical rehabilitation program [9].

**Figure 1:**
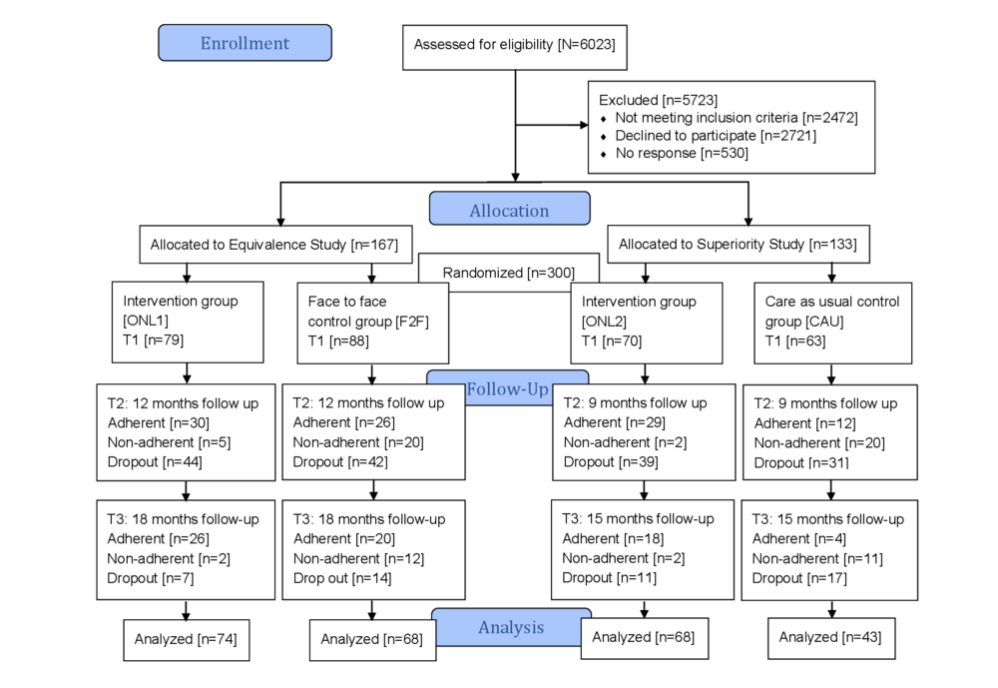
CONSORT (Consolidated Standards of Reporting Trials) flowchart for the Curriculum Hannover. CAU: care as usual, F2F: face-to-face, ONL: online.

Rehabilitants were questioned during their stay by a member of the social services staff and asked whether they wanted to take advantage of an offer for aftercare therapy. If they said yes, they were informed about the option of participating in the study and about the study conditions. If they agreed to participate, the rehabilitants who had a potential F2F therapy offering within a 45-minute radius of their place of residence were randomly assigned to either F2F therapy or online therapy within the *equivalence study* (Figure 1) [9]. Those without therapy offerings in their vicinity were randomly assigned to online therapy or no therapy within the so-called *superiority study*.

Overall, 300 rehabilitants were assigned to one of the study arms: For the equivalence study, 167 rehabilitants were randomized to the online therapy (ONL1) or the F2F therapy (Figure 1). For the superiority study, 133 rehabilitants were randomly assigned to online therapy (ONL2) or the control group (CAU) [9].

### Study Participants

After providing informed consent, 300 patients were included in the study (see Figure 1). All patients had participated in rehabilitation treatment [10] lasting between 1 week and 8 weeks (median 5 weeks; mean 5.11, SD 1.30 weeks) in 1 of 4 clinics [9].

Individuals had been admitted to rehabilitative treatment due to psychosomatic diagnoses according to the International Classification of Disease, 10th revision (ICD-10) manual (F20.0-F61 and G43.1), with the most frequent diagnoses being F32.1 Depressive episode (55/300), F33.1 Recurrent depressive disorder (87/300), and Adjustment disorders (33/300). At the conclusion of their rehabilitation, all individuals were encouraged to maintain their rehabilitation treatment success [9,10,24,25]. Individuals received the following treatment recommendations: medication check-up (270/300); psychotherapy and psychological counseling (246/300); therapeutic support including physio-, ergo-, or dietary therapy (171/300); aftercare (162/300); addiction counseling (32/300); stationary treatment (4/300); hours-wise reintegration into the job (31/300); diagnostics (30/300); return to work support (21/300); self-help group (5/300); and other recommendations (180/300).

The sample had a mean age of 50.27 (SD 9.71; range 25-67; median 52; mode 58) years. More women participated in the study (199/300, 66.3%), 51.0% (153/300) were partnered/married, and 75.7% (227/300) were employed.

### Implementation of Therapy

The therapy in the usual F2F contact was based on the concept from Curriculum Hannover [24,25], an aftercare treatment following the medical rehabilitation. Apart from the admission and final interviews, which were each a 50-minute individual interview, therapy took place over 25 weekly 90-minute group sessions with 8-10 participants.

The online aftercare was carried out according to the same concept but had technical peculiarities due to the digital format [9,10,24,25]: The participants were instructed in advance on how to use the video platform by means of a learning video; this included, among other things, the rules for communication in the virtual group room as well as instructions for regularly checking the internet connection. The psychotherapists prepared for the special features of the new format through training courses geared to equip them to build a therapeutic relationship in a targeted manner. Also, they were instructed to teach their patients how to use the video platform. As a part of the group therapy, psychoeducation was conveyed using PowerPoint presentations or a whiteboard. Handouts or homework could also be distributed [9].

Under study conditions, CAU therapy meant that no standardized therapy measures were initiated. For these patients, only whether they independently took up treatments was assessed.

### Statistical Analysis

#### Comparisons

Differences between the intervention and control groups in terms of dropout and adherence (research question 1) were tested with frequency analyses (chi-squared). Patients’ reasons as to why they did not adhere (research question 2 and the sample description) were examined using descriptive analyses without calculating any statistics.

To test what factors were related to completing the different study arms (research question 3), *t* tests, chi-squared tests, and logistic regression analyses (determining odds ratios [ORs]) were conducted.

A multiple analysis of variance (MANOVA) with post-hoc Bonferroni tests was performed testing whether the subjective evaluation of the online and F2F therapy would differ with regard to relationship satisfaction, success satisfaction, and satisfaction with therapy (research question 4). This was also followed up by logistic regression analyses.

To test whether the subjective evaluation of the online and F2F therapy differed with regard to relationship satisfaction, success satisfaction, and satisfaction with therapy, a MANOVA was performed (research question 5) taking expectations into account. All analyses were run using SPSS 26.

#### Power

To determine the minimum required sample size through a priori analysis for obtaining a significant medium effect size, we used PADD 11 software and G*Power 3 software (Psychonomic Society Inc, Düsseldorf, Germany) [26,27]. With regard to the superiority study and based on the assumption of an effect of the intervention with a Cohen *d*>0.60 (>8 points difference in the primary endpoint), a total of 90 subjects (45 per study arm) was needed to conduct the study at an alpha <.05 and power >0.80. Concerning the equivalence study and based on the assumption of an equivalence margin of Cohen *d*<0.29 (<4 points difference in the primary endpoint), a total of 410 subjects (205 per study arm) was planned for the study to test at an alpha <.05 and power >.80 [24,25].

#### Data Exclusion

Inclusion criteria were an indication for psychosomatic therapy (indication is determined by the procedure described in the German Pension Insurance's framework concept for rehabilitation therapy [10]) and access to a standard PC, tablet, or smartphone with internet access (DSL or LTE) [9].

The exclusion criteria were also based on the framework concept for rehabilitation therapy of the German Pension Insurance and included employability <3 hours/day on the general labor market, drawing or applying for an old-age pension of at least two-thirds of the full-time pension, and drawing a benefit that is paid regularly until the start of a pension due to old age. Furthermore, patients with acute psychosomatic disorders were excluded [9,10].

The survey was conducted during rehabilitation by means of questionnaire measures at baseline (T1), 9 (superiority study) or 12 (equivalence study) months after the end of rehabilitation (after completion of the therapy intervention; T2), and 15 (superiority study) or 18 (equivalence study) months after the end of rehabilitation (T3). The reason for the postponed survey in the equivalence study was the wait time for an aftercare place in the F2F therapy.

The study participants were asked to fill out the T2 and T3 questionnaires by email and were reminded 2 weeks and 4 weeks later, respectively. The questionnaires were filled out digitally via the platform soscisurvey.de. At T2 and T3, the patients were asked whether they really followed the study protocol in terms of adhering to the study arm and its treatment as they were assigned [10].

#### Survey Instruments

The following items were analyzed in this longitudinal study [9]: sociodemographic information on gender, age, marital status, educational level, employment status, and income level.

To measure different aspects of mental health, the Hamburg Modules for the Assessment of Psychosocial Health in Clinical Practice (HEALTH-49) [28] were used for the purpose of this study. Individuals were asked to answer different modules, such as their feelings during the past 2 weeks or symptoms during the past 2 weeks, assessing various aspects of mental health. Items belonging to modules A and C were measured on a 5-point Likert scale from 1 (“not at all”) to 5 (“very much”). Modules B, E, and F were also measured on a 5-point Likert scale from 1 (“never”) to 5 (“always”). Module D was also measured on a 5-point Likert scale from 1 (“not true”) to 5 (“very true”).

Subjective employment prognosis [29] was measured via 3 items. The first item assessed whether individuals believed they would work until retirement. This item was measured on a 5-point Likert scale from 1 (“sure”) to 5 (“not at all”). The second item assessed whether individuals perceived their overall earning capacity was at risk due to their health status. This item was measured on a 2-point Likert scale from 1 (“no”) to 2 (“yes”). The third and last item evaluated whether individuals were thinking about applying for pension due to health limitations, and this was measured on a 3-point Likert scale: 1 (“no”), 2 (“yes”), and 3 (“I have already submitted a pension application”).

In addition, 3 items of the Work Ability Index (WAI) [30] were implemented to provide an assessment of the ability to work. The first item, “If you rate your best ever work ability as 10 points: How many points would you then give for your current work ability (0 means you are currently unable to work)?” was assessed on an 11-point Likert scale from 0 (“completely unable to work”) to 10 (“currently the best working capacity”). In addition, the 2 items “How would you rate your current work ability in terms of physical job demands?” and “How would you rate your current work ability in terms of mental work demands?” were both measured on a 5-point Likert scale from 1 (“very poorly”) to 5 (“very good”).

Additionally, the subscale hope of improvement of the standardized Patient Questionnaire on Therapy Expectation and Evaluation (PATHEV) was used to assess their expectation of improvement in symptoms after therapy [31]. Participants were asked 4 items, which were measured on a 5-point Likert scale from 1 (“completely disagree”) to 5 (“completely agree”).

Further, the therapeutic relationship was assessed by means of the Helping Alliance Questionnaire (HAQ) using 2 subscales [32]: Subscale 1 focuses on relationship satisfaction (8 items), and subscale 2 focuses on success satisfaction (3 items). All items were measured on a 6-point Likert scale from 1 (“very inaccurate”) to 6 (“very accurate”).

Satisfaction with rehabilitation was evaluated using a standardized 8-item questionnaire [33]. Items were assessed on a 5-point Likert scale with different anchors used for the 8 items. Additionally, a self-developed questionnaire was used to assess participation in outpatient measures and therapy in the CAU group.

## Results

### Differences Between Groups

The 300 participants were divided into 2 RCTs: equivalence study (n=167) and superiority study (n=133; Table 1). All 300 participants completed the questionnaire at baseline (T1). The number of participants who completed the questionnaires at T2 and T3 is shown in Table 1 indicating retention rates.

**Table 1:** Study retention rates for the follow-up measurements in the equivalence study (online therapy vs face-to-face; n=167) and the superiority study (online therapy vs care as usual; n=133).

Time point	Equivalence study					Superiority study			
	ONL1^a^ (n=79), n (%)	F2F^b^ (n=88), n (%)	χ^2^_1_	*P* value	ONL2^c^ (n=70), n (%)		CAU^d^ (n=63), n (%)	χ^2^_1_	*P* value
T2^e^	35 (44)	46 (52)	1.06	.30	31 (44)		32 (51)	0.56	.453
T3^f^	28 (35)	32 (36)	0.02	.90	20 (29)		15 (24)	0.39	.533

^a^ONL1: online therapy in the equivalence study.

^b^F2F: face-to-face therapy.

^c^ONL2: online therapy in the superiority study.

^d^CAU: care as usual.

^e^T2: 12-month follow-up measurement for the equivalence study; 9-month follow-up measurement for the superiority study.

^f^T3: 18-month follow-up measurement for the equivalence study; 15-month follow-up measurement for the superiority study.

At T2, 2 participants partially completed the questionnaire and did not respond to questions regarding the outcome of treatment. These 2 patients were still labeled as completers as they responded to the questionnaire in general (and were included in all analyses except for the therapeutic relationship and satisfaction). All participants who completed the questionnaire at T3 also completed the questionnaire at T2. No differences between the ONL1 and F2F groups were found either at T2 or T3. Similarly, no differences between the ONL2 and CAU groups were found at either T2 or T3.

These results partially answer research question 1: There was no significant difference between the online therapy groups (ONL1, ONL2) and the control groups (F2F, CAU) regarding the percentage of patients who completed the questionnaires (identified as completers). To also test whether patients in the online therapy groups differed regarding their adherence to the assigned therapy, we further investigated the nonadherence rates across all patients at all time points. Differences per measurement point were tested within the 167 patients assigned to the equivalence study and the 133 patients in the superiority study (Table 2).

**Table 2:** Study retention (number and percentage of patients who completed the questionnaires; completers) and dropout rates (no completion of the questionnaires), as well as adherence to the assigned therapy for both the follow-up measurement points T2 and T3, by equivalence study (n=167) and superiority study (n=133).

Time points			Equivalence study									Superiority study				
			ONL1^a^ (n=79), n (%)		F2F^b^ (n=88), n (%)		χ^2^_2_		*P* value		ONL2^c^ (n=70), n (%)		CAU^d^ (n=63), n (%)	χ^2^_2_		*P* value
**T2^e^**																
	Adherent completers	30 (38)		26 (30)		8.87		.012		29 (41)			12 (19)	22.38	<.001	
	Nonadherent completers	5 (6)		20 (23)						2 (3)			20 (32)			
	Dropouts	44 (56)		42 (48)						39 (56)			31 (49)			
**T3^f^**																
	Adherent completers	26 (33)		20 (23)		–^g^		–^g^		18 (26)			4 (6)	–^g^	–^g^	
	Nonadherent completers	2 (3)		12 (14)						2 (3)			11 (18)			
	Dropouts	51 (65)		56 (64)						50 (71)			48 (76)			

^a^ONL1: online therapy in the equivalence study.

^b^F2F: face-to-face therapy in the equivalence study.

^c^ONL2: online therapy in superiority study.

^d^CAU: care as usual in the superiority study.

^e^T2: 12-month follow-up measurement for the equivalence study; 9-month follow-up measurement for the superiority study.

^f^T3: 18-month follow-up measurement for the equivalence study; 15-month follow-up measurement for the superiority study.

^g^Test statistic could not be computed because the cell frequencies were too small.

Patients randomized into the F2F and CAU control groups were much more likely to show nonadherent behavior (not adhering to the therapy to which the study participants were assigned) in comparison with the online therapy groups. In both studies, these differences in dropout and nonadherence were statistically significant (Table 2). More patients dropped out from the study at T3, and the nonadherence rates were lower than at T2 (Table 2). Descriptively, the highest percentage of study participants retained in the study were in the F2F group (32/88, 36%), whereas patients randomized to online therapy were retained at a slightly smaller percentage (ONL1: 28/79, 35% and ONL2: 20/70, 29%; Table 2). The highest risk for dropout from the study was in the CAU group (which did not receive any therapy within the study). However, the differences could not be analyzed statistically due to small cell sizes at T3.

Summarizing findings regarding research question 1, differences between the online therapy groups (ONL1, ONL2) and the control groups (F2F, CAU) occurred regarding both the percentage of patients who completed the questionnaires (completers) and patients who adhered to the assigned condition. These differences were statistically significant at T2 and only descriptive at T3.

### Exploring Reasons for Nonadherence

To study research question 2, at T2, nonadherent patients were asked, using a set of predefined answers, why they did not adhere to the therapy to which they were assigned, and each patient could select the answers that most applied. Among patients randomized to ONL1 and identified as nonadherent (5/79), 2 patients answered that they felt the rehabilitation was already sufficient to meet their therapy goals and therefore did not see any further need to participate in the aftercare therapy. One patient indicated that s/he would not perceive the therapy as useful. One patient indicated a lack of motivation to attend the therapy, whereas another patient cited time constraints.

Of the patients randomized into the F2F group who were nonadherent (20/88), about one-third (7/20, 35%) cited the unavailability of such treatment, could not find therapy at all, or would have had to wait too long. There were 5 patients who answered that the location of the therapy would be too far away. Another 2 patients replied that they would not perceive the therapy as useful, while 2 patients indicated that the therapy would be too much of a strain. One patient indicated that s/he could not motivate her/himself to attend the therapy.

The 2 nonadherent participants from the 70 participants in the ONL2 group both answered that they did not participate in the therapy due to time constraints and other reasons. Of the 63 patients assigned to CAU, 20 were nonadherent: Of those, 16 patients participated independently from this study in an outpatient treatment. Another 2 underwent inpatient treatment, 14 received drug therapy, and 2 attended a self-help group.

Thus, to address research question 2, patients had different reasons for not adhering to the assigned therapy related to the study arm. Summarizing the online treatments (ONL1 and ONL2), the 7 nonadherent patients indicated no motivation (n=1), lack of understanding of the benefits of aftercare (n=1), no time (n=2), rehabilitation goals already achieved (n=2).

### Comparisons Between Retained Patients and Those Who Dropped Out at T2 and T3

To investigate research question 3 and the differences between patients who completed the study and those who dropped out (not retained in the study), we compared sociodemographic variables within the study arms at T2 (Table 3) and T3 (Multimedia Appendix 1). At T2, only gender was related with study dropout in the CAU group: While the completer group consisted of 59% (19/32) women, the dropouts were female (26/31, 84%) with a higheer likelihood. Thus, within the CAU group, it seemed to be more difficult for women to be retained in the study than it was for men. However, such gender differences did not appear in any other group at a significant level (see Table 3).

**Table 3:** The differences between patients who completed the study and those who dropped out at T2 (12-month follow-up measurement for the equivalence study; n=167; 9-month follow-up measurement for the superiority study; n=133).

Variables		Equivalence study ONL1^a^				Equivalence study F2F^b^				Superiority study ONL2^c^				Superiority study CAU^d^		
		Completers^e^ (n=35)	Dropouts^f^ (n=44)	*P* value	Completers^e^ (n=46)		Dropouts^f^ (n=42)	*P* value	Completers^e^, (n=31)		Dropouts^f^ (n=39)	*P* value	Completers^e^ (n=32)		Dropouts^f^ (n=31)	*P* value
Age (years), mean (SD)		50.23 (9.36)	50.98 (9.09)	.72	51.48 (8.88)		49.50 (10.26)	.34	51.32 (10.15)		48.97 (10.36)	.35	51.75 (9.32)		47.58 (10.65)	.10
Gender (female), n (%)		27 (77)	26 (59)	.09	27 (59)		24 (57)	.88	19 (61)		31 (80)	.09	19 (59)		26 (84)	.031
Marital status (Married), n (%)		24 (67)	25 (57)	.29	23 (50)		23 (55)	.66	16 (52)		17 (44)	.50	18 (56)		11 (36)	.10
**Educational level, n (%)**											.					
	Elementary school	18 (51)	23 (52)	.90	19 (41)		22 (52)	.58	16 (52)		26 (67)	.24	16 (50)		11 (36)	.38
	High school	6 (17)	10 (23)		14 (30)		10 (24)		2 (7)		4 (10)		5 (16)		9 (29)	
	College and above	9 (26)	9 (21)		13 (28)		10 (24)		13 (42)		9 (23)		11 (34)		10 (32)	
	Other	2 (7)	2 (5)		0		0		0		0		0		1 (3)	
Employment status (employed), n (%)		27 (77)	34 (77)	.99	34 (74)		29 (69)	.61	27 (87)		30 (77)	.28	23 (72)		23 (74)	.84
**Income status (€^g^), n (%)**																
	<1500	8 (23)	11 (25)	.52	13 (28)		15 (36)	.65	11 (36)		13 (33)	.38	9 (28)		9 (29)	.67
	1500-3000	17 (49)	16 (36)		23 (50)		17 (41)		9 (29)		17 (44)		15 (47)		17 (55)	
	>3000	10 (29)	17 (39)		10 (22)		10 (24)		11 (36)		9 (56)		8 (25)		5 (16)	

^a^ONL1: online therapy in the equivalence study.

^b^F2F: face-to-face therapy in the equivalence study.

^c^ONL2: online therapy in the superiority study.

^d^CAU: care as usual in the superiority study.

^e^Completers: study participants who completed the questionnaires.

^f^Dropouts: study participants who dropped out from the study and did not complete the questionnaires.

^g^A currency exchange rate of €1=US $1.16 is applicable.

To investigate research question 3 regarding T3, the same analyses were performed with the second follow-up measurement (Multimedia Appendix 1). Age and marital status emerged as significantly differentiating between completers and dropouts in the CAU group: In the completers group, patients were, on average, 7 years older than patients in the dropout group. Moreover, for patients randomized into the CAU group, married patients were more likely to be retained in the study, whereas single patients were much more likely to drop out. However, similar age and marital status differences did not appear on a significant level in any other group (see Multimedia Appendix 1). To summarize the results on research question 3, the only factors that were related to the completion of the different study arms at T2 and T3 were age, gender, and marital status. However, these were only bivariate analyses, and in the following sections, we investigate the interrelations in a multivariate approach.

### Predicting Study Retention in All 4 Groups

To further examine research question 3 and to investigate whether the intervention group (Model 0), sociodemographic variables (Model 1), health-related and psychosocial variables, as well as work ability at baseline (Model 2) were interrelated with the completion of the study at T2 and T3 (Table 4), logistic regression analyses were performed. This was done using dummy coding for patients who remained in the study at T2 and T3 as 1 vs those who dropped out as 0.

After matching the results from the comparisons between the 4 groups, the study arm (ie, intervention group to which the patients were randomly assigned) was not significantly related with retention when comparing the 3 groups receiving a treatment (ONL1, F2F, ONL2) with the CAU control group (Intervention [ONL vs F2F] in Multimedia Appendix 2). After controlling for the sociodemographic variables (Model 2 T2, Model 2 T3, with no variable being significant), 2 characteristics emerged for study retention at T2 and T3: Patients who reported lower depressiveness scores at T1 (better mental health; T2: OR 0.51, 95% CI 0.30-0.85; T3: OR 0.45, 95% CI 0.25-0.79) were more likely to be retained in the study, and patients with higher self-efficacy (T2: OR 0.50, 95% CI 0.30-0.83; T3: 0.57, 95% CI 0.33-0.99) were more likely to drop out. Additionally, at T3, higher social support seemed to make it more likely that patients dropped out: the less social support patients reported, the more they remained in the study (but this was not significant at T2; T2: OR 0.79, 95% CI 0.54-1.15; T3: OR 0.67, 95% CI 0.44-0.99; Table 4).

These findings from the 3 intervention groups (Multimedia Appendix 2) matched the findings from all 4 groups (Table 4). Remarkably, with all 4 groups, being married emerged as significant in Model 2 when controlling for the health-related variables, psychosocial variables, and work ability at baseline, indicating a suppressor effect: Only when parts of the variance were explained by depressiveness, self-efficacy, and social support, those who were single were more likely to remain in the study. Additionally, on a marginal/descriptive level, findings from these analyses were validated, with retention higher in the F2F group (Model 0 T2: OR 1.06, 95% CI 0.56-2.03; Model 1 T2: OR 1.05, 95% CI 0.54-2.05; Model 2 T2: OR 1.14, 95% CI 0.57-2.28; Model 1 T3: OR 1.93, 95% CI 0.92-4.07; Model 2 T3: OR 2.10, 95% CI 0.96-4.59), for older participants (Model 1 T2: OR 1.02, 95% CI 0.99-1.04; Model 2 T2: OR 1.02, 95% CI 0.99-1.04; Model 1 T3: OR 1.03, 95% CI 0.99-1.06; Model 2 T3: OR 1.03, 95% CI 1.00-1.06), and for married participants (Model 1 T2: OR 1.55, 95% CI 0.94-2.57; Model 2 T2: OR 1.80, 95% CI 1.05-3.08; Model 1 T3: OR 1.47, 95% CI 0.85-2.52; Model 2 T3: OR 1.78, 95% CI 1.00-3.20; Table 4).

Summarizing findings regarding research question 3, the following factors were (partially) related with the completion of the different study arms at T2 and T3: age, marital status, depressiveness, self-efficacy, and social support.

**Table 4:** Logistic regression models predicting study retention with all patients: online therapy in the equivalence study (ONL1), face-to-face therapy in the equivalence study (F2F), online therapy in superiority study (ONL2), care as usual in the superiority study (CAU).

Variables		Model 0 T2^a^		Model 1 T2			Model 2 T2			Model 1 T3^b^			Model 2 T3	
		OR^c^ (95% CI)	*P* value	OR (95% CI)	*P* value	OR (95% CI)		*P* value	OR (95% CI)		*P* value	OR (95% CI)		*P* value
**Intervention group**														
	ONL1 vs CAU	0.77 (0.39- 1.50)	.44	0.75 (0.38-1.50)	.42	0.74 (0.37-1.52)		.43	1.79 (0.83-3.86)		.14	1.82 (0.82-4.03)		.14
	F2F vs CAU	1.06 (0.56-2.03)	.86	1.05 (0.54-2.05)	.88	1.14 (0.57-2.28)		.71	1.93 (0.92-4.07)		.08	2.10 (0.96-4.59)		.06
	ONL2 vs CAU	0.77 (0.39-1.52)	.45	0.75 (0.37-1.53)	.43	0.78 (0.38-1.64)		.52	1.29 (0.58-2.88)		.54	1.32 (0.57-3.07)		.52
Age		N/A^d^	N/A	1.02 (0.99-1.04)	.21	1.02 (0.99-1.04)		.28	1.03 (0.99-1.06)		.06	1.03 (1.00-1.06)		.08
**Gender**														
	Female	N/A	N/A	1	N/A	1		N/A	1		N/A	1		N/A
	Male	N/A	N/A	1.15 (0.70-1.90)	.58	1.22 (0.72-2.07)		.45	1.00 (0.58-1.71)		.99	1.01 (0.57-1.78)		.98
**Marital status**														
	Unmarried	N/A	N/A	1	N/A	1		N/A	1		N/A	1		N/A
	Married	N/A	N/A	1.55 (0.94-2.57)	.09	1.80 (1.05-3.08)		.03	1.47 (0.85-2.52)		.17	1.78 (1.00-3.20)		.05
**Educational level**														
	Elementary school	N/A	N/A	1	N/A	1		N/A	1		N/A	1		N/A
	High school	N/A	N/A	0.89 (0.48-1.66)	.72	1.02 (0.53-1.98)		.95	0.85 (0.43-1.70)		.65	0.96 (0.46-2.00)		.91
	College and above	N/A	N/A	1.42 (0.81-2.49)	.22	1.63 (0.90-2.96)		.11	1.28 (0.70-2.35)		.42	1.50 (0.79-2.85)		.22
	Other	N/A	N/A	0.75 (0.12-4.82)	.76	0.68 (0.10-4.62)		.70	2.23 (0.18-8.19)		.83	1.07 (0.15-7.78)		.95
**Employment status**														
	Unemployed	N/A	N/A	1	N/A	1		N/A	1		N/A	1		N/A
	Employed	N/A	N/A	1.13 (0.62-2.05)	.70	1.13 (0.60-2.12)		.71	1.51 (0.77-2.95)		.23	1.68 (0.82-3.45)		.16
**Income status (€^e^)**														
	<1500	N/A	N/A	1	N/A	1		N/A	1		N/A	1		N/A
	1500-3000	N/A	N/A	0.96 (0.53-1.76)	.91	0.91 (0.48-1.72)		.78	1.04 (0.54-1.99)		.92	1.06 (0.53-2.12)		.87
	>3000	N/A	N/A	0.76 (0.37-1.56)	.45	0.78 (0.36-1.66)		.51	0.58 (0.26-1.28)		.18	0.63 (0.27-1.46)		.28
Somatoform complaints		N/A	N/A	N/A	N/A	0.97 (0.67-1.38)		.84	N/A		N/A	0.91 (0.61-1.35)		.64
Depressiveness		N/A	N/A	N/A	N/A	0.55 (0.35-0.87)		.01	N/A		N/A	0.56 (0.37-0.92)		.02
Phobic fear		N/A	N/A	N/A	N/A	1.15 (0.81-1.64)		.42	N/A		N/A	1.30 (0.88-1.93)		.18
Mental well-being		N/A	N/A	N/A	N/A	1.47 (0.90-2.40)		.13	N/A		N/A	1.63 (0.95-2.80)		.07
Interactional difficulties		N/A	N/A	N/A	N/A	1.21 (0.87-1.68)		.26	N/A		N/A	1.29 (0.90-1.86)		.16
Self-efficacy		N/A	N/A	N/A	N/A	0.57 (0.37-0.89)		.01	N/A		N/A	0.52 (0.32-0.85)		.01
Activity and participation		N/A	N/A	N/A	N/A	1.25 (0.85-1.83)		.26	N/A		N/A	1.06 (0.70-1.60)		.80
Social stress		N/A	N/A	N/A	N/A	0.85 (0.60-1.21)		.37	N/A		N/A	0.71 (0.48-1.06)		.09
Social support		N/A	N/A	N/A	N/A	0.79 (0.58-1.08)		.14	N/A		N/A	0.68 (0.48-0.96)		.03
Work ability index		N/A	N/A	N/A	N/A	0.98 (0.76-1.27)		.89	N/A		N/A	0.96 (0.72-1.27)		.75
Nagelkerke		.007	.646	.043	.62	.119		.05	.067		.272	.197		.004

^a^T2: 12-month follow-up measurement for the equivalence study; 9-month follow-up measurement for the superiority study.

^b^T3: 18-month follow-up measurement for the equivalence study; 15-month follow-up measurement for the superiority study.

^c^OR: odds ratio.

^d^N/A: not applicable.

^e^A currency exchange rate of €1=US $1.16 is applicable.

### Expectations for Improvement From Face-to-Face and Online Therapy

To test research question 4, improvement expectation from therapy was measured at baseline among the ONL1, F2F, and ONL2 groups to evaluate any differences. We conducted a MANOVA controlling for age, gender, and marital status. The results showed significant differences in improvement expectation based on the study arm (*F*_2,228_=3.13, *P*=.05, Eta²=.027) and based on dropout rates (*F*_1,228_=5.68, *P*=.02, Eta²=.024), after controlling for age (*P*=.96; Eta²<.001), gender (*P*=.89; Eta²<.001), and marital status (*P*=.91; Eta²<.001).

**Figure 2:**
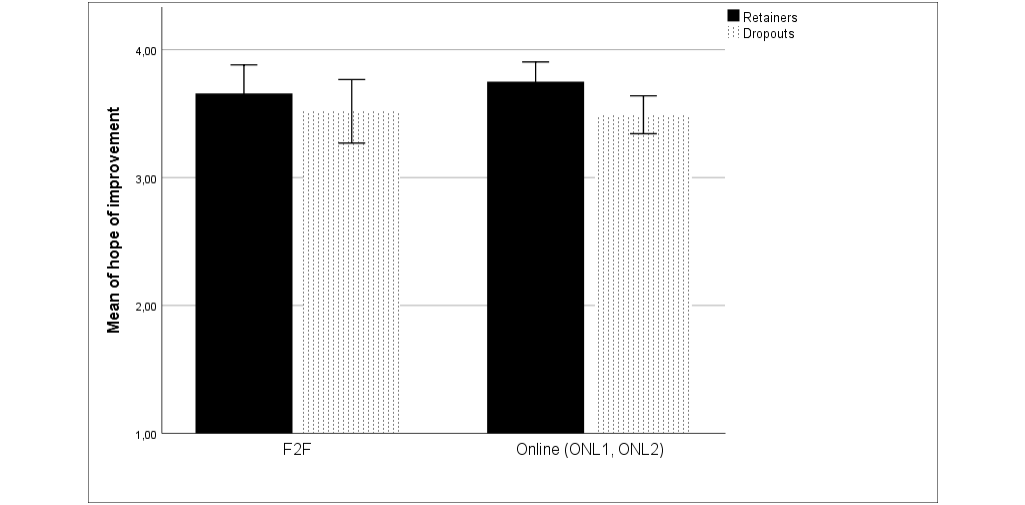
Mean scores for improvement expectation during therapy among patients who completed the questionnaires and those who dropped out at T2, in the face-to-face (F2F) and combined online groups (ONL1+ONL2).

When conducting post-hoc comparisons of differences between patients who completed the questionnaires and those who did not at T2, the 2 online groups (ONL1+ONL2) were contrasted with the F2F group. We found a significant difference (*F*_1,147_=5.74, *P*=.02): The mean score for improvement expectation from therapy among the online therapy groups was significantly higher among patients who completed the questionnaire than among those who did not complete the questionnaire at T2 (see Figure 2). The difference among the F2F therapy group did not show any significant effects (*F*_1,86_=0.716, *P*=.40).

When further exploring the post-hoc comparisons on improvement expectation between patients who completed the questionnaires and those who dropped out at T3 among online (ONL1+ONL2) vs F2F therapy, the combined online therapy group emerged as significant (*F*_1,147_=5.74, *P*=.04). The mean score for improvement expectation was higher among patients who completed the questionnaire (mean 3.76, SD 0.61) than among patients who dropped out (mean 3.52, SD 0.68). The differences among patients receiving F2F therapy was not significant (*F*_1,83_=0.486, *P*=.49).

### Predicting Study Retention With Improvement Expectations in the Intervention Groups

Logistic regression analyses were performed as described with patients in the ONL1, F2F, and ONL2 groups and including improvement expectation (Multimedia Appendix 2). Improvement expectation was significant when included in the analyses: Those who had higher improvement expectations were also those who were more likely to complete study questionnaires (Model 2 T2: OR 1.64, 95% CI 1.08-2.50; Model 2 T3: OR 1.59, 95% CI 1.01-2.51; Multimedia Appendix 2).

To answer research question 4, we can summarize that improvement expectations did differ between the online therapy groups (ONL1, ONL2) and the control groups (F2F, CAU). Positive expectation was also significantly related with completion of the questionnaire at T2 and T3 after controlling for other variables: the more the patients in all groups expected, the more likely they were to be retained in the study.

### Evaluation of the Different Therapies With Regard to Different Satisfaction Aspects

To test research question 5, whether subjective evaluations of the online and F2F therapies differed with regard to relationship satisfaction, success satisfaction, and satisfaction with therapy, we conducted a MANOVA. The therapeutic relationship included 2 aspects: relationship satisfaction (HAQ1) and success satisfaction (HAQ2). Both items, together with satisfaction with the treatment, were analyzed using a MANOVA to evaluate overall effects, and then we tested for group differences (study arm ONL1, F2F, ONL2, and patients with high vs low expectations) using Bonferroni post-hoc tests. Only the intervention groups explained significant proportions of the variance (*F*_Roy’s Largest Root;3,76_=2.665, *P*=.05, Eta²=.095). Expectation and the interaction of intervention and expectation were not significant (*P*>.45). The means and SDs as well as summary statistics are reported in Table 5 along with the *F* tests of the individual test variables, indicating that the overall effect was based on satisfaction with the therapy. Within this variable, the post-hoc tests revealed that differences between groups only existed between online and F2F groups, but not between the 2 online groups.

**Table 5:** Statistical results of the therapeutic relationship and satisfaction with the therapy in patients with low expectations (below a median of 3.50, n=31) vs high expectations (above the median, n=52).

Variables			ONL1^a^ (n=30), mean (SD)		F2F^b^ (n=24), mean (SD)		ONL2^c^ (n=29), mean (SD)		CAU^d^		*F*_group_		*P* value		Eta²
**Relationship satisfaction (HAQ1^e^)**															
	Low expectations	2.63 (0.67)		2.84 (0.95)		2.52 (1.01)		N/A^f^		0.937		.40		.02	
	High expectations	2.90 (1.67)		2.98 (1.11)		2.38 (1.18)									
**Success satisfaction (HAQ2)**															
	Low expectations	2.45 (0.61)		2.65 (0.83)		2.51 (1.18)		N/A		0.697		.50		.018	
	High expectations	2.46 (1.60)		2.87 (1.26)		2.21 (1.17)									
**Satisfaction with therapy^g^**															
	Low expectations	3.16 (0.41)		2.80 (0.65)		3.50 (0.43)		N/A		3.907		.024		.092	
	High expectations	3.33 (0.94)		2.69 (1.06)		3.22 (0.91)									

^a^ONL1: online therapy in the equivalence study.

^b^F2F: face-to-face therapy in the equivalence study.

^c^ONL2: online therapy in the superiority study.

^d^CAU: care as usual in the superiority study.

^e^HAQ: Helping Alliance Questionnaire.

^f^N/A: not applicable because not measured in the CAU group.

^g^Effect for expectation was not significant. Post-hoc tests revealed a significant difference between satisfaction and therapy: ONL1 > F2F (*P*=.05); ONL2 > F2F (*P*=.03).

To summarize findings regarding research question 5, the subjective evaluation of the online and F2F therapies differed based on relationship satisfaction and success satisfaction and specifically with satisfaction with the therapy. Patients randomized into the online therapy were significantly more satisfied with their treatment than patients in the F2F group. Relationship satisfaction and success satisfaction were equally high in the online and the F2F treatments.

## Discussion

In medical internet research, it has been shown that outpatient psychotherapeutic treatment after rehabilitation treatment is an important factor in ensuring the sustainability of treatment effects [1-9,24,25]. At the same time, research has demonstrated the benefits of online psychotherapy in comparison with F2F psychotherapy on site [34]. For the first time, this study showed the direct comparison of psychotherapeutic aftercare delivered online versus F2F with CAU at more than 1-year follow-up (ie, 15 months and 18 months after baseline) in the field of psychosomatic therapy regarding adherence and dropout rates. This is important due to the fact that individually tailored online and F2F therapies have greater interventional effects than standard therapy programs [3,5-9].

### Principal Findings

Of 300 patients participating in the T1 measurement point, 167 were assigned to the equivalence study because they had F2F psychotherapeutic aftercare available, and 133 were assigned to the superiority study because of the unavailability of aftercare options. Within both groups, the patients were randomized to the online psychotherapeutic aftercare or the comparator group. Retention rates in the online groups were equal in all groups. However, retaining the patients for periods of 18 months (ONL1, F2F) and 15 months (ONL2, CAU) was rather difficult: While after 9 months or 12 months, 56% of the patients in the online therapy dropped out from the study, a further 9%-15% (ie, 65%-71% in total) had dropped out after 15 months or 18 months. Whether the difference in dropout between the F2F group (48%-64%) and the CAU group (49%-76%) was due to the longer time frame of the follow-up measurement point or to the different conditions remains unclear at this point.

In addition, nonadherence rates were tested, and the F2F and CAU groups were much more likely to show nonadherent behavior in comparison with the online therapy groups: While the online groups were only nonadherent at 3%-6%, the F2F group was much more likely to be nonadherent, at 14%-23%. Self-reported reasons were mainly unavailability of the treatment, waiting times that were too long, and a location that was too far away.

The highest nonadherence occurred in the CAU condition, at 18%-32%. Reported reasons for this nonadherence were that patients participated in an outpatient psychotherapeutic aftercare, inpatient psychotherapy, drug therapy, or a self-help group independently from this study. These alternatives to just keep waiting could, of course, be regarded as good for the patient and as functional behavior. In contrast, dropout appears to have been unfavorable, as the patients’ condition may have worsened making them unable to participate in the study while experiencing no or inappropriate support. Thus, determining how to meet the needs of the patients is of the highest priority to prevent dropout and dysfunctional nonadherence.

Accordingly, differences between completers and dropouts were tested in bivariate analyses; age, gender, and marital status were significant in the CAU group. Women, younger individuals, and people who were single had a more difficult time remaining in the study than men, older individuals, and partnered patients. When testing this with all groups combined and statistically controlling for these group differences, only marital status was significant. However, this was merely the case when the psychological factors were included as well. It turned out that patients who indicated more depressiveness, more self-efficacy, and more social support were also more likely to drop out. This indicates that reducing symptoms in patients who are in greater need of treatment is difficult.

Furthermore, symptom reduction requires corresponding communication that builds tolerance against perceiving instant improvements but rather investing in active participation in treatment. This relates to the findings regarding improvement expectations: the more improvements patients expect due to the aftercare treatment, the more likely they are to also remain in the study after controlling for the aforementioned factors. Thus, working on those expectations right at the beginning of the therapy is imperative, especially if patients report high levels of depressiveness.

At the same time, it should be kept in mind that all the study participants had finished an intensive inpatient rehabilitation treatment and those still suffering from depression might have had a chronic or therapy-resistant medical condition. This might have led to the failure of aftercare and resulted in dropouts, which calls even more for individualized treatments addressing chronic depressiveness and the risks of relapse.

In contrast, the finding that patients with more self-efficacy and higher social support were more likely to drop out is noteworthy; this could be interpreted as unmet needs and expectations. At the same time, patients with high self-efficacy and social support might feel more capable to find better support or treatment. On the other hand, those with low self-efficacy and low social support seem to be a good fit for the online and F2F therapies provided in this study. More attention should be paid to patients with low expectations who appear difficult to cater to.

Regarding satisfaction with the aftercare, the participants in the online aftercare groups indicated higher satisfaction values than the participants in the F2F group. This can be explained by the advantage of requiring less time for commuting to the therapist [8] and the absence of the fear of stigmatization in online therapies [15]. Further studies are required for a precise clarification.

The fact that participants in the online aftercare groups rated the therapeutic relationship better than the participants in the F2F aftercare group might be due to the so-called online disinhibition effect, implying that people are more open to share their emotions and conflicts in a virtual space [35].

### Limitations

This study was designed to investigate online therapies, compared with F2F therapy and CAU, in terms of symptom improvement. One main limitation was that the CAU group was not assessed regarding their expectations, due to practical and ethical considerations. Another limitation was the fact that dropouts were not specifically re-assessed because this was not planned within the study protocol [9].

However, further prospective and randomized studies are necessary to investigate the actual acceptance of online therapy opportunities and the prevention of dropout from (online) therapies and measurements [3-8,34,35]. Additionally, testing tailoring of the programs to the expectations and resources of the patients, specifically with regard to dropout and nonadherence, could provide additional insight. In the future, clinical trial registration should be prospective instead of retrospective.

Another noteworthy limitation is that 6023 patients were recruited but only 300 patients (about 5%) took part in the study. While reasons may vary from local and individual factors, it may also be the case that the program was interesting and fitting only for a very small subgroup of the addressed population.

In the future, the program should be designed in a way so that it better matches a larger proportion of the sample. Ideally, co-creative or co-design strategies that involve the target group could help, although this is typically very time and resource consuming.

### Comparison With Prior Work

In this study, more patients dropped out than in other online intervention studies [6,13], which might be related with the longer follow-up period in this study. However, in their systematic review, Brown et al [11] did not find the intended duration of the program to be significant. More work is needed on the dose-response, along with testing whether the right length and intensity of therapy are related with lower dropout rates.

In the study by Zarski et al [3], 14% of the variance in treatment adherence could be explained by the variables of the HAPA model [22]. In our study, sociodemographic variables explained 4%-5% of questionnaire completion rates, which increased to 17%-20% when including social-cognitive variables related to the HAPA and additional health-related characteristics. While this percentage of the variance may appear small, one has to bear in mind that the predictor variables were assessed at baseline and dropouts at 15 or 18 months later, whereas in the study by Zarski et al [3], only a baseline measure and adherence 7 weeks later were analyzed.

In the systematic review by Brown et al [11], the duration of the interventions ranged between 3 and 20 weeks, with the majority lasting 8 weeks (n=25), 6 weeks (n=22), or 10 weeks (n=8). Thus, our study evaluated the intervention over a relatively long time frame, and more studies like this are required in the future to replicate our findings with larger samples.

Consistent with previous studies [6], dropout was less prevalent on a descriptive level in the group with more human feedback and less feedback filtered by the online delivery (ie, in the F2F group relative to all other groups). While previous studies [11] uncovered factors such as hardware or technical issues, this could also be assumed for this study, too, but few patients in the 2 online therapy groups actually reported this explicitly. On the other hand, the online therapy clearly overcame previously reported problems such as lack of time and work commitments [11,14], as well as commuting challenges to the physical intervention site [4,7,12,36].

Other problems such as disinterest and a diminishing desire to participate, perceptions that no further need for treatment would be required, feeling better after only a few modules, and perceiving the program as noneffective were found across all groups [11]. However, disappointment due to group assignment can be assumed, especially in the CAU group, and may be a reason for dropout and nonadherence [11].

The finding that poor health [6,11,17,20] could be related to poor adherence was also found in this study. However, this was only found with regard to depressiveness—the main symptom for assigning patients to psychosomatic rehabilitation treatment and its aftercare.

Other interrelations we revealed in our study also matched those in previous studies. For instance, higher education was (partially) related to lower dropout, probably because more self-reflection and eloquence make it easier to make use of the therapy [4,20,21]. No clear evidence regarding employment could be found in previous research [14], and our data supported this finding. While in previous studies, age was found to be related to the willingness to participate and remain in online research [14], we also found that younger patients were at a greater risk of dropping out [16].

Our finding that women were more likely to drop out from the study if randomized to the CAU group also matched previous findings (eg, [19]). If this could be attributed to the participating women being less technologically open and self-efficacious and less able to overcome technical problems [20], there would be a need for training and more adequate support. However, if this is related to family duties such as caring for children or other family members as found in previous studies [8,17,20], the online therapy might bring benefits both in terms of avoiding commuting times or eliminating the risk of leaving children or family members unattended at home. Nevertheless, this was not explicitly assessed in this study and calls for future research.

Online therapy might also bring the risk of multitasking at home and creating no clear detachment from family duties when spending time in therapy. Such patients may be just one wall away from family responsibilities, and this may also relate to difficulties explaining to family members that no disturbances are allowed. This could be addressed in terms of good planning, for instance, by having a babysitter both for attending a F2F treatment at a therapy site and, while ensuring confidentiality, when attending the online therapy at home.

Marital status and social support were revealed in our study as being significantly related to remaining in online interventions as has been shown in previous research [8,14,17]. However, an opposite pattern to previous studies [14,20] was found: Marital status was beneficial but more social support was not. Maybe a family member stepping in when problems, such as increasing family duties, made it more likely for individuals to remain in the therapy and the study, despite the difficulty. This underscores the importance of partnership or family for therapy adherence. On the contrary, dropout for those patients with high social support may be due to having perceived a mismatch in their expectations but then they got the help to find alternatives (while the partner or family individual is not able to do so). However, these assumptions need to be researched further, in more detail, and systematically.

Matching previous findings, higher treatment expectancy regarding the treatment efficacy was related to greater study retention and adherence [14,20]. Remarkably, contrary to previous studies [3,14], in our study, self-efficacy was found to be directly related to dropout. As mentioned, this may be related to the study design and other factors relating to alternative treatment usage and self-help behavior. Thus, more work is needed to investigate this further.

### Conclusions

This study showed that there are many different factors correlating with adherence to and dropout from online and F2F therapies. These variables should be addressed when allocating patients to their therapies and treating mental disorders.

Special focus should be given to women, younger patients, unpartnered patients, less educated patients, patients with more depressiveness symptoms, and those with fewer expectations. Tailored approaches should support these patients by meeting their needs and building optimistic expectations.

## Appendix

Multimedia Appendix 1 The differences between patients who completed questionnaires at T3 and those who dropped out from the study at T2 and T3.

Multimedia Appendix 2 Logistic regression models predicting completion of the questionnaire at T2 and T3 with only the patients receiving a therapy (ON1, F2F, ONL2).

Multimedia Appendix 3 CONSORT-EHEALTH checklist (V 1.6.1).

## Abbreviations

CAU: care as usual
F2F: face-to-face
HAPA: health action process approach
HAQ: Helping Alliance Questionnaire
HEALTH-49: Hamburg Modules for the Assessment of Psychosocial Health in Clinical Practice
ICD-10: International Classification of Disease, 10th revision
MANOVA: multiple analysis of variance
ONL1: Curriculum-Hannover-Online therapy in equivalence study
ONL2: Curriculum-Hannover-Online therapy in superiority study
PATHEV: Patient Questionnaire on Therapy Expectation and Evaluation
RCT: randomized controlled trial
T1: baseline
T2: 12-month follow-up measurement for the equivalence study; 9-month follow-up measurement for the superiority study
T3: 18-month follow-up measurement for the equivalence study; 15-month follow-up measurement for the superiority study.
WAI: Work Ability Index

## References

[1] Lecomte T, Potvin S, Corbière M, Guay S, Samson C, Cloutier B, Francoeur A, Pennou A, Khazaal Y (2020). Mobile apps for mental health issues: meta-review of meta-analyses. JMIR Mhealth Uhealth.

[2] Wu A, Scult MA, Barnes ED, Betancourt JA, Falk A, Gunning FM (2021). Smartphone apps for depression and anxiety: a systematic review and meta-analysis of techniques to increase engagement. NPJ Digit Med.

[3] Zarski A, Berking M, Reis D, Lehr D, Buntrock C, Schwarzer R, Ebert DD (2018). Turning good intentions into actions by using the Health Action Process Approach to predict adherence to internet-based depression prevention: secondary analysis of a randomized controlled trial. J Med Internet Res.

[4] Erbe D, Eichert H, Riper H, Ebert DD (2017). Blending face-to-face and internet-based interventions for the treatment of mental disorders in adults: systematic review. J Med Internet Res.

[5] Lindhiem O, Bennett CB, Rosen D, Silk J (2015). Mobile technology boosts the effectiveness of psychotherapy and behavioral interventions: a meta-analysis. Behav Modif.

[6] Torous J, Lipschitz J, Ng M, Firth J (2020). Dropout rates in clinical trials of smartphone apps for depressive symptoms: A systematic review and meta-analysis. J Affect Disord.

[7] Karin E, Dear BF, Heller GZ, Crane MF, Titov N (2018). "Wish you were here": examining characteristics, outcomes, and statistical solutions for missing cases in web-based psychotherapeutic trials. JMIR Ment Health.

[8] Skea ZC, Newlands R, Gillies K (2019). Exploring non-retention in clinical trials: a meta-ethnographic synthesis of studies reporting participant reasons for drop out. BMJ Open.

[9] Dahmen A, Gao L, Keller F, Lehr D, Becker P, Lippke S (2021). Wirksamkeit webbasierter psychotherapeutische Nachsorge nach psychosomatischer Rehabilitation ? ein Test in zwei Randomized Controlled Trials. Die Rehabilitation (forthcoming).

[10] Deutsche Rentenversicherung.

[11] Brown M, O'Neill N, van Woerden H, Eslambolchilar P, Jones M, John A (2016). Gamification and adherence to web-based mental health interventions: a systematic review. JMIR Ment Health.

[12] Buyruk Genç A, Amanvermez Y, Zeren SG, Erus SM (2019). Early separations: Dropout from online and face-to-face counseling. Pegem Eğitim ve Öğretim Dergisi.

[13] Marks IM, Kenwright M, McDonough M, Whittaker M, Mataix-Cols D (2004). Saving clinicians' time by delegating routine aspects of therapy to a computer: a randomized controlled trial in phobia/panic disorder. Psychol Med.

[14] Beatty L, Binnion C (2016). A systematic review of predictors of, and reasons for, adherence to online psychological interventions. Int J Behav Med.

[15] Weightman M (2020). Digital psychotherapy as an effective and timely treatment option for depression and anxiety disorders: Implications for rural and remote practice. J Int Med Res.

[16] Karin E, Crane MF, Dear BF, Nielssen O, Heller GZ, Kayrouz R, Titov N (2021). Predictors, outcomes, and statistical solutions of missing cases in web-based psychotherapy: methodological replication and elaboration study. JMIR Ment Health.

[17] Linardon J, Fuller-Tyszkiewicz M (2020). Attrition and adherence in smartphone-delivered interventions for mental health problems: A systematic and meta-analytic review. J Consult Clin Psychol.

[18] Cai Z, Fan X, Du J (2017). Gender and attitudes toward technology use: A meta-analysis. Computers & Education.

[19] Retzer L, Reindl R, Zauter S, Richter K (2021). Bevorzugen Frauen Face-to-Face-Beratung bei Insomnie?. Somnologie.

[20] Al-Asadi AM, Klein B, Meyer D (2014). Posttreatment attrition and its predictors, attrition bias, and treatment efficacy of the anxiety online programs. J Med Internet Res.

[21] Schmidt ID, Forand NR, Strunk DR (2019). Predictors of dropout in internet-based cognitive behavioral therapy for depression. Cognit Ther Res.

[22] Schwarzer R, Lippke S, Luszczynska A (2011). Mechanisms of health behavior change in persons with chronic illness or disability: the Health Action Process Approach (HAPA). Rehabil Psychol.

[23] Davies F, Shepherd HL, Beatty L, Clark B, Butow P, Shaw J (2020). Implementing web-based therapy in routine mental health care: systematic review of health professionals' perspectives. J Med Internet Res.

[24] Kobelt A, Nickel L, Grosch EV, Lamprecht F, Künsebeck HW (2004). [Participation in psychosomatic outpatient care after in-patient rehabilitation]. Psychother Psychosom Med Psychol.

[25] Kobelt A, Grosch E (2005). Indikation zur ambulanten Nachsorge (Curriculum Hannover) in der Psychosomatischen Rehabilitation. Psychotherapeut.

[26] Faul F, Erdfelder E, Lang A, Buchner A (2007). G*Power 3: A flexible statistical power analysis program for the social, behavioral, and biomedical sciences. Behavior Research Methods.

[27] NCSS Statistical Software.

[28] Rabung S, Harfst T, Kawski S, Koch U, Wittchen H, Schulz H (2009). [Psychometric analysis of a short form of the "Hamburg Modules for the Assessment of Psychosocial Health" (HEALTH-49)]. Z Psychosom Med Psychother.

[29] Mittag O, Meyer T, Glaser-Möller N, Matthis C, Raspe H (2006). [Predicting gainful employment in a population sample of 4225 statutory pension insurance members covering a prognostic period of five years using a brief subjective prognostic employment scale (SPE Scale)]. Gesundheitswesen.

[30] Tuomi K, Ilmarinen J, Jahkola A, Katajarinne L, Tulkki A (1998). Work Ability Index, 2nd revised edition.

[31] Schulte D (2005). Messung der Therapieerwartung und Therapieevaluation von Patienten (PATHEV). Zeitschrift für Klinische Psychologie und Psychotherapie.

[32] Nübling R, Kraft M, Henn J, Kriz D, Lutz W, Schmidt J, Wittmann W, Bassler M (2017). [Testing the psychometric properties of the Helping Alliance Questionnaire (HAQ) in different health care settings]. Psychother Psychosom Med Psychol.

[33] Schmidt J, Nübling R, Brähler E, Schumacher J, Strauß B (2002). ZUF-8. Fragebogen zur Messung der Patientenzufriedenheit. Diagnostische Verfahren in der Psychotherapie.

[34] Andersson G, Cuijpers P, Carlbring P, Riper H, Hedman E (2014). Guided internet-based vs. face-to-face cognitive behavior therapy for psychiatric and somatic disorders: a systematic review and meta-analysis. World Psychiatry.

[35] Mehta VS, Parakh M (2015). Web based interventions in psychiatry: an overview. Int J Ment Health Psychiatry.

[36] Lippke S, Dahmen A, Gao L, Guza E, Nigg CR (2021). To what extent is internet activity predictive of psychological well-being?. PRBM.

