# Supplementary material for: Adherence With Online Therapy vs Face-to-Face Therapy and With Online Therapy vs Care as Usual: Secondary Analysis of Two Randomized Controlled Trials
Source: J Med Internet Res. 2021 Nov 3;23(11):e31274. doi: 10.2196/31274 (PMC8600425; doi:10.2196/31274)
Supplement: Multimedia Appendix 1 [file jmir_v23i11e31274_app1.docx]

Multimedia Appendix 1. The differences between patients who completed questionnaires at T3 and those who dropped out from the study at T2 and T3.

| Variables | | Equivalence study (n = 167) | | | | | | Superiority study (n = 133) | | | | | |
| --- | --- | --- | --- | --- | --- | --- | --- | --- | --- | --- | --- | --- | --- |
|  |  | ONL1 | | | F2F | | | ONL2 | | | CAU | | |
|  |  | C^a^ (n=28) | D^b^ (n=51) | *P* | C^a^ (n=32) | D^b^ (n=56) | *P* | C^a^ (n=20) | D^b^ (n=50) | *P* | C^a^ (n=15) | D^b^ (n=48) | *P* |
|  | |  |  |  |  |  |  |  |  |  |  |  |  |
| Age (years), mean (SD) | | 50.25 (9.07) | 50.86 (9.29) | .778 | 51.13 (9.17) | 50.20 (9.84) | .664 | 53.60 (7.69) | 48.58 (10.86) | .064 | 55.47 (7.82) | 47.90 (10.17) | .010 |
| Female, n (%) | | 22 (78.6) | 31 (60.8) | .108 | 18 (56.3) | 33 (58.9) | .807 | 13 (65.0) | 37 (74.0) | .451 | 9 (60.0) | 36 (75.0) | .262 |
| Marital status (Married), n (%) | | 19 (67.9) | 30 (58.8) | .429 | 13 (40.6) | 29 (51.8) | .313 | 10 (50.0) | 23 (46.0) | .762 | 12 (80.0) | 17 (35.4) | .002 |
| Education, n (%) | |  |  | .893 |  |  | .691 |  | . | .552 |  |  | .913 |
|  | Elementary school | 14 (50.0) | 27 (52.9) |  | 16 (50.0) | 25 (44.6) |  | 11 (55.0) | 31 (62.0) |  | 6 (40.0) | 21 (43.8) |  |
|  | High school and equal | 5 (17.9) | 11 (21.6) |  | 7 (21.9) | 17 (30.4) |  | 1 (5.0) | 5 (10.0) |  | 4 (26.7) | 10 (20.8) |  |
|  | College and above | 7 (25.0) | 11 (21.6) |  | 9 (39.1) | 14 (60.9) |  | 8 (40.0) | 14 (28.0) |  | 5 (33.3) | 16 (33.3) |  |
|  | Other | 2 (7.1) | 2 (3.9) |  | 0 | 0 |  | 0 | 0 |  | 0 | 1 (2.1) |  |
| Employed, n (%) | | 23 (82.1) | 38 (74.5) | .439 | 24 (75) | 39 (69.6) | .592 | 18 (90.0) | 39 (78.0) | .243 | 12 (80.0) | 34 (70.8) | .485 |
| Income status, n (%) | |  |  | .485 |  |  | .412 |  |  | .592 |  |  | .348 |
|  | < 1,500 | 5 (17.9) | 14 (27.5) |  | 10 (31.3) | 18 (32.1) |  | 8 (40.0) | 16 (32.0) |  | 3 (20.0) | 15 (31.3) |  |
|  | 1,500 - 3,000 | 14 (50.0) | 19 (37.3) |  | 17 (53.1) | 23 (41.1) |  | 8 (40.0) | 18 (36.0) |  | 7 (46.7) | 25 (52.1) |  |
|  | > 3,000 | 9 (32.1) | 18 (35.3) |  | 5 (15.6) | 15 (26.8) |  | 4 (20.0) | 16 (32.0) |  | 5 (33.3) | 8 (16.7) |  |

^a^C: Study participants who completed the questionnaires (retainers).

^b^D: Study participants who dropped out from the study and did not complete the questionnaires.
