# Supplementary material for: Adherence With Online Therapy vs Face-to-Face Therapy and With Online Therapy vs Care as Usual: Secondary Analysis of Two Randomized Controlled Trials
Source: J Med Internet Res. 2021 Nov 3;23(11):e31274. doi: 10.2196/31274 (PMC8600425; doi:10.2196/31274)
Supplement: Multimedia Appendix 2 [file jmir_v23i11e31274_app2.docx]

Multimedia Appendix 2. Logistic regression models predicting completion of the questionnaire at T2 and T3 with only the patients receiving a therapy (ON1, F2F, ONL2).

| Variables | | Model 1 T2 | | Model 2 T2 | | Model 1 T3 | | Model 2 T3 | |
| --- | --- | --- | --- | --- | --- | --- | --- | --- | --- |
|  |  | OR [95%CI] | *P* | OR [95%CI] | *P* | OR [95%CI] | *P* | OR [95%CI] | *P* |
|  | |  |  |  |  |  |  |  |  |
| Intervention (ONL vs. F2F) | | 1.41 [0.81, 2.45] | .224 | 1.58 [0.87, 2.88] | .136 | 1.26 [0.70, 2.26] | .441 | 1.43 [0.75, 2.72] | .274 |
| Age | | 1.01 [0.98, 1.04] | .418 | 1.01 [0.98, 1.04] | .564 | 1.02 [0.99, 1.05] | .225 | 1.02 [0.98, 1.05] | .308 |
| Gender | |  |  |  |  |  |  |  |  |
|  | Female | 1 |  | 1 |  | 1 |  | 1 |  |
|  | Male | 0.95 [0.54, 1.66] | .181 | 1.02 [0.56, 1.84] | .959 | 0.95 [0.52, 1.71] | .860 | 1.00 [0.52, 1.89] | .988 |
| Marital status | |  |  |  |  |  |  |  |  |
|  | Unmarried | 1 |  | 1 |  | 1 |  | 1 |  |
|  | Married | 1.47 [0.84, 2.60] | .181 | 1.71 [0.92, 3.15] | .088 | 1.15 [0.63, 2.07] | .655 | 1.32 [0.69, 2.52] | .406 |
| Educational level | |  |  |  |  |  |  |  |  |
|  | Elementary school | 1 |  | 1 |  | 1 |  | 1 |  |
|  | Higher school and equal | 1.17 [0.58, 2.56] | .663 | 1.28 [0.60, 2.73] | .520 | 0.83 [0.38, 1.79] | .629 | 0.86 [0.37, 1.96] | .711 |
|  | College and above | 1.73 [0.91, 3.26] | .093 | 2.06 [1.04, 4.12] | .040 | 1.34 [0.67, 2.60] | .385 | 1.60 [0.77, 3.30] | .207 |
|  | Other | 1.33 [0.17, 10.31] | .788 | 1.17 [0.14, 9.64] | .886 | 2.36 [0.29, 19.31] | .424 | 1.80 [0.20, 16.03] | .599 |
| Employment status | |  |  |  |  |  |  |  |  |
|  | Unemployed | 1 |  | 1 |  | 1 |  | 1 |  |
|  | Employed | 1.30 [0.65, 2.57] | .457 | 1.48 [0.69, 3.16] | .315 | 1.56 [0.74, 3.29] | .241 | 1.83 [0.80, 4.17] | .150 |
| Income status, n (%) Euro/ month | |  |  |  |  |  |  |  |  |
|  | < 1,500 | 1 |  | 1 |  | 1 |  | 1 |  |
|  | 1,500 - 3,000 | 0.95 [0.48, 1.90] | .885 | 0.91 [0.43, 1.92] | .796 | 1.05 [0.51, 2.15] | .904 | 0.97 [0.44, 2.14] | .938 |
|  | > 3,000 | 0.69 [0.31, 1.55] | .373 | 0.71 [0.30, 1.72] | .451 | 0.54 [0.23, 1.27] | .156 | 0.53 [0.21, 1.40] | .185 |
| Somatoform complaints | |  |  | 1.03 [0.67, 1.59] | .901 |  |  | 0.99 [0.62, 1.59] | .971 |
| Depressiveness | |  |  | 0.51 [0.30, 0.85] | .010 |  |  | 0.45 [0.25, 0.79] | .005 |
| Phobic fear | |  |  | 1.20 [0.79, 1.82] | .389 |  |  | 1.41 [0.90, 2.19] | .134 |
| Mental well-being | |  |  | 1.41 [0.79, 2.51] | .245 |  |  | 1.77 [0.95, 3.30] | .070 |
| Interactional difficulties | |  |  | 1.36 [0.91, 2.03] | .136 |  |  | 1.33 [0.87, 2.02] | .190 |
| Self-efficacy | |  |  | 0.50 [0.30, 0.83] | .008 |  |  | 0.57 [0.33, 0.99] | .045 |
| Activity and participation | |  |  | 1.19 [0.76, 1.86] | .445 |  |  | 1.24 [0.77, 2.01] | .378 |
| Social stress | |  |  | 0.91 [0.59, 1.38] | .654 |  |  | 0.76 [0.48, 1.19] | .231 |
| Social support | |  |  | 0.79 [0.54, 1.15] | .219 |  |  | 0.67 [0.44, 0.99] | .054 |
| Work ability index | |  |  | 1.05 [0.77, 1.44] | .759 |  |  | 0.90 [0.64, 1.26] | .530 |
| Expectation | |  |  | 1.64 [1.08, 2.50] | .022 |  |  | 1.59 [1.01, 2.51] | .045 |
| Nagelkerke | | .042 | .72 | .167 | .014 | .050 | .551 | .197 | .004 |
